# High Incidence of Severe Combined Immunodeficiency Disease in Saudi Arabia Detected Through Combined T Cell Receptor Excision Circle and Next Generation Sequencing of Newborn Dried Blood Spots

**DOI:** 10.3389/fimmu.2018.00782

**Published:** 2018-04-16

**Authors:** Hamoud Al-Mousa, Ghadah Al-Dakheel, Amal Jabr, Fahd Elbadaoui, Mohamed Abouelhoda, Mansoor Baig, Dorota Monies, Brian Meyer, Abbas Hawwari, Majed Dasouki

**Affiliations:** ^1^Department of Pediatrics, King Faisal Specialist Hospital & Research Center, Riyadh, Saudi Arabia; ^2^Department of Genetics, Research Center, King Faisal Specialist Hospital & Research Center, Riyadh, Saudi Arabia; ^3^College of Medicine, Alfaisal University, Riyadh, Saudi Arabia; ^4^Saudi Human Genome Project, King Abdulaziz City for Science and Technology, Riyadh, Saudi Arabia; ^5^Department of Biostatistics, Epidemiology & Scientific Computing (BESC), King Faisal Specialist Hospital & Research Center, Riyadh, Saudi Arabia; ^6^King Abdullah International Medical Research Center (KAIMRC), Ministry of National Guard Health Affairs, Riyadh, Saudi Arabia

**Keywords:** severe combined immunodeficiency, dried blood spot, immunodeficiency, newborn screening, Saudi, T-cell receptor excision circle, Enlite, SCID

## Abstract

Severe combined immunodeficiency disease (SCID) is the most severe form of primary immunodeficiency disorders (PID). T-cell receptor excision circle (TREC) copy number analysis is an efficient tool for population-based newborn screening (NBS) for SCID and other T cell lymphopenias. We sought to assess the incidence of SCID among Saudi newborn population and examine the feasibility of using targeted next generation sequencing PID gene panel (T-NGS PID) on DNA isolated from dried blood spots (DBSs) in routine NBS programs as a mutation screening tool for samples with low TREC count. Punches from 8,718 DBS collected on Guthrie cards were processed anonymously for the TREC assay. DNA was extracted from samples with confirmed low TREC count, then screened for 22q11.2 deletion syndrome by real-time polymerase chain reaction and for mutations in PID-related genes by T-NGS PID panel. Detected mutations were confirmed by Sanger sequencing. Sixteen out of the 8,718 samples were confirmed to have low TREC copy number. Autosomal recessive mutations in *AK2, JAK3, and MTHFD1* were confirmed in three samples. Two additional samples were positive for the 22q11.2 deletion syndrome. In this study, we provide evidence for high incidence of SCID among Saudi population (1/2,906 live births) and demonstrate the feasibility of using T-NGS PID panel on DNA extracted from DBSs as a new reliable, rapid, and cost-effective mutation screening method for newborns with low TREC assay, which can be implemented as part of NBS programs for SCID.

## Introduction

Severe combined immunodeficiency disease (SCID) is considered a medical emergency since it is the most severe form of primary immunodeficiency disorders (PID). SCID is a genetically heterogeneous group of over 20 monogenic inherited inborn errors of the immune system characterized by lack of T lymphocytes development from the thymus, in addition to deficiencies of B cells, NK cells, or both in some forms. Impaired cellular and humoral immunity makes affected infants susceptible to bacterial, viral, fungal, and opportunistic infections and results in death within the first 2 years of life. Infants with SCID can be rescued with hematopoietic stem cell transplantation (HSCT) and for some forms by using gene therapy or enzyme replacement therapy (1–4).

Severe combined immunodeficiency disease meets newborn screening (NBS) disease criteria, as affected infants are asymptomatic at birth, disease is lethal without treatment, and outcome significantly improve if early management is offered (5–7). HSCT is the most effective curative treatment and the outcome is influenced by age at diagnosis and clinical status of patients at presentation (8, 9). High (95%) overall survival was observed among infants undergoing transplantation in the first month of life in comparison to those treated after 3 months of age (70%) (3–7).

Excision and splicing of the DNA encoding the T-cell antigen receptor genes are required during normal T cell development and differentiation to produce T cells with diverse antigen specificities. During T cell receptor rearrangement, deleted DNA fragments circulate without further replication in dividing cells. T-cell receptor excision circle (TRECs) is an excellent marker of recently formed T cells (10). In 2005, Chan and Puck first described the TREC assay as an excellent tool for population-based NBS for SCID (11). TREC copy number can be determined from Guthrie card dried blood spots (DBSs) using real-time quantitative polymerase chain reaction (RT-qPCR) (12).

In 2008, the TREC assay-based NBS for SCID was first implemented in WI, USA (13). Universal SCID NBS was then recommended in 2010 by the US Advisory Committee on Heritable Disorders in Newborns and Children. Currently, most states provide NBS for SCID using the TREC assay and several pilot studies are ongoing in Europe and Asia (14–18). The results of screening three million newborns showed a SCID frequency of 1/58,000 children and a higher overall frequency (1/7,300) of significant forms of T cell lymphopenia (19). SCID incidence is expected to be higher in populations with high rates of consanguinity (20–22).

Depending on how you set the cut limit, TREC assay can also identify non-SCID immunodeficiencies with profound decrease in circulating naïve T-cells including: 22q11 deletion syndrome, trisomy21, CHARGE syndrome, Kabuki syndrome, Ataxia telangiectasia, *DOCK8-*related autosomal recessive hyper IgE syndrome, Cartilage hair hypoplasia, Nijmegen breakage syndrome, Schimke immune-osseous dysplasia, preterm infants, and idiopathic lymphopenia in addition to other disorders such as vascular leakage, chylous effusions, congenital leukemia, and congenital heart diseases, which cause T cell loss (19, 23–26).

Most causes of monogenic SCID have an autosomal recessive mode of inheritance and hence they are expected to be more common in areas with high rates of consanguineous marriages (21, 27, 28). Consanguineous marriages are common in Saudi Arabia with an overall incidence of 60% (29). In this study, we have conducted a pilot TREC-based NBS to assess the incidence of SCID and other combined immunodeficiencies associated with low TREC among the Saudi population and examine the clinical utility of a targeted next generation sequencing (NGS) PID gene panel as a confirmatory diagnostic tool for mutation screening of Guthrie card DBS with low TREC count.

## Materials and Methods

### Ethical Approval

All research involving Guthrie card (DBS) samples was approved by the institutional review board at King Faisal Specialist Hospital & Research Center (KFSHRC), RAC:2130-027. The ethical board waived informed consent since samples were anonymized. This work was supported by the Kingdom of Saudi Arabia National Science, Technology and Innovation Plan’s Strategic Technologies Grant KACST: 13-BIO 755-20 under the King Abdulaziz City for Science and Technology (Riyadh, Kingdom of Saudi Arabia).

### Screened Samples

In Saudi Arabia, NBS officially began in 2005. Currently, there are three NBS programs that provide screening for about 50% of all (570,000) newborns (30). The Newborn Screening & Biochemical Genetics Laboratory at King Faisal Specialist Hospital and Research Centre (KFSHRC) receives samples from all provinces of Saudi Arabia and screens about 80–90,000 live births every year. DBS specimens were obtained from Guthrie cards collected over a 12-month period (November 10, 2015–November 30, 2016). Within 1–3 days after completing the routine NBS tests, DBS samples were randomly selected with an average of 1,000 samples every month. Prior to anonymization, gestational age and birth weight data were collected. Large punches from selected DBSs were anonymized and coded to prevent any possibility of tracing the identity of the newborn behind the sample. The anonymized samples were then punched and processed for the TREC assay and potential additional second tier analyses.

### TREC Assay

The Enlite™ Neonatal TREC assay is an FDA approved, high throughput (96 or 384 well) based assay (Perkin Elmer, Turku, Finland). The details of this assay had been described previously (14, 18, 31). Briefly, the assay utilizes 2-plex amplification of TREC and beta-actin (ACTB) in the same reaction for each specimen. It uses a combination of end-point polymerase chain reaction (PCR)-based deoxyribonucleic acid amplification and time-resolved fluorescence resonance energy transfer (TR-FRET)-based detection. Calibrator and control samples are used to calculate TREC and ACTB copies and for monitoring the assay performance. Unlike all other routine NBS assays, which use a standard 3.2 mm punch, for the Enlite assay uses a 1.5 mm DBS punch from each sample into a PCR-plate directly, which is then incubated with 10 µL elution buffer (5× Phire^®^ buffer mixed with elution diluent) followed by combined DNA amplification and hybridization, and signal measurement using the VICTOR Enlite fluorometer. And the EnLite™ workstation software was used to interpret test results based on two separate calibration curves, which use blanks and DBS calibrators A–C. The response, corrected counts, are fitted against the ArcSinh transformed concentrations (copies/μL) using the unweighted linear regression. The test quality control is based on three kit control results interpretations. The initial manufacturer provided cutoffs were as follows: TREC positive: ≥36/μL, TREC negative: ≥36 copies/μL, and Beta-actin (ACTB) copy of ≥56 copies/μL. The test result is valid when Beta-actin copy of ≥56 copies/μL. Since by study design, no clinical follow-up of screen positive cases was possible and to ensure the identification of all newborns with classical SCID and possibly other combined immunodeficiencies with low TREC, we elected to use the manufacturer recommended cutoff at 36 copies/μL. To test the validity of this cutoff limit, a previously collected Guthrie card samples from nine molecularly confirmed patients with atypical AK2 deficiency were utilized for assay validation and setting the cutoff limit. Triplicate TREC copy number analysis was run in each sample except one, which was run in singlicate. The mean and median TREC copy number was 13.3 and 13.5, respectively, while the range was 0–28 copies/μL.

### DNA Extraction

The MasterPure™ Complete DNA & RNA Purification Kit (epicenter, Madison, WI, USA) was used to isolate DNA from DBSs as recommended by the manufacturer. Briefly, six DBS punches were treated with proteinase K followed by adding a cell lysis solution and protein precipitation. Recovered DNA was re-suspended in 20–30 µl of TE buffer.

### Targeted NGS PID Panel (T-NGS PID)

Ion AmpliSeq Designer software (Life Technologies, Carlsbad, CA, USA) was used for the design and synthesis of a multiplexed gene panel encompassing 265 PID genes. The list of PID genes screened for is provided in Table S1 in Supplementary Material. Primers were optimized to provide amplicons (200 bp) with at least 90% coverage of coding sequence and a minimum of 10 bp flanking regions of associated introns. Ten nanograms of extracted DNA samples were sufficient to generate the Ion Proton AmpliSeq library. Details of library preparation, NGS, data processing, bioinformatics analysis, workflow of detecting point mutations, and copy number variants (CNVs) were previously described (28). The assay has an overall sensitivity of 96% for single nucleotide variants (SNVs) and 92% for detecting Indels. CNV analyses have a sensitivity of 100% for detecting large homozygous deletions (28).

### Sanger Sequencing

Identified mutations by T-NGS PID panel were confirmed by standard Sanger sequencing. Details of PCR, primers design, and sequencing was described previously (32, 33). Sequence data were aligned against the reference GenBank sequences and examined for variation. Novel mutations were compared against local Saudi Genomic as well as non-Saudi population databases. Pathogenicity of novel mutations was determined based on population allele frequency and *in silico* prediction tools.

### Chr.22q11.2 Copy Number/Deletion Analysis

Copy number-based assays (RT-qPCR and single nucleotide polymorphism-microarrays) were used to detect this deletion. To test gDNA from NBS DBS samples with low TRECs, we used a TaqMan labeled *TBX1* real-time qPCR copy number assay (ThermoScientific).^1^ This assay is run as a duplex real-time PCR of target gDNA (*TBX1*) as well the reference gene (*RNase P* H1 RNA gene) both of which are normally present as diploid copies. The comparative CT (ΔΔCT) method was then used to calculate the number of copies of the target sequence in each test sample [measures the CT difference (ΔCT) between target and reference sequences, then compares the ΔCT values of test samples to a calibrator sample(s) known to have two copies of the target sequence]. The copy number of the target is calculated to be two times the relative quantity.^2^ Samples and controls (known 22q11.2 deletion and normal) were run in duplicates in each 96-well plate. Samples with abnormal copy number for *TBX1* (22q11.2 deletion) were repeated in duplicates as well. CopyCaller™ Software (ThermoFisher) was used to calculate target (*TBX1*) as well as reference (*RNAse P*) copy number in all samples.

## Results

### Neonatal Guthrie Card Samples

**Figure 1:**
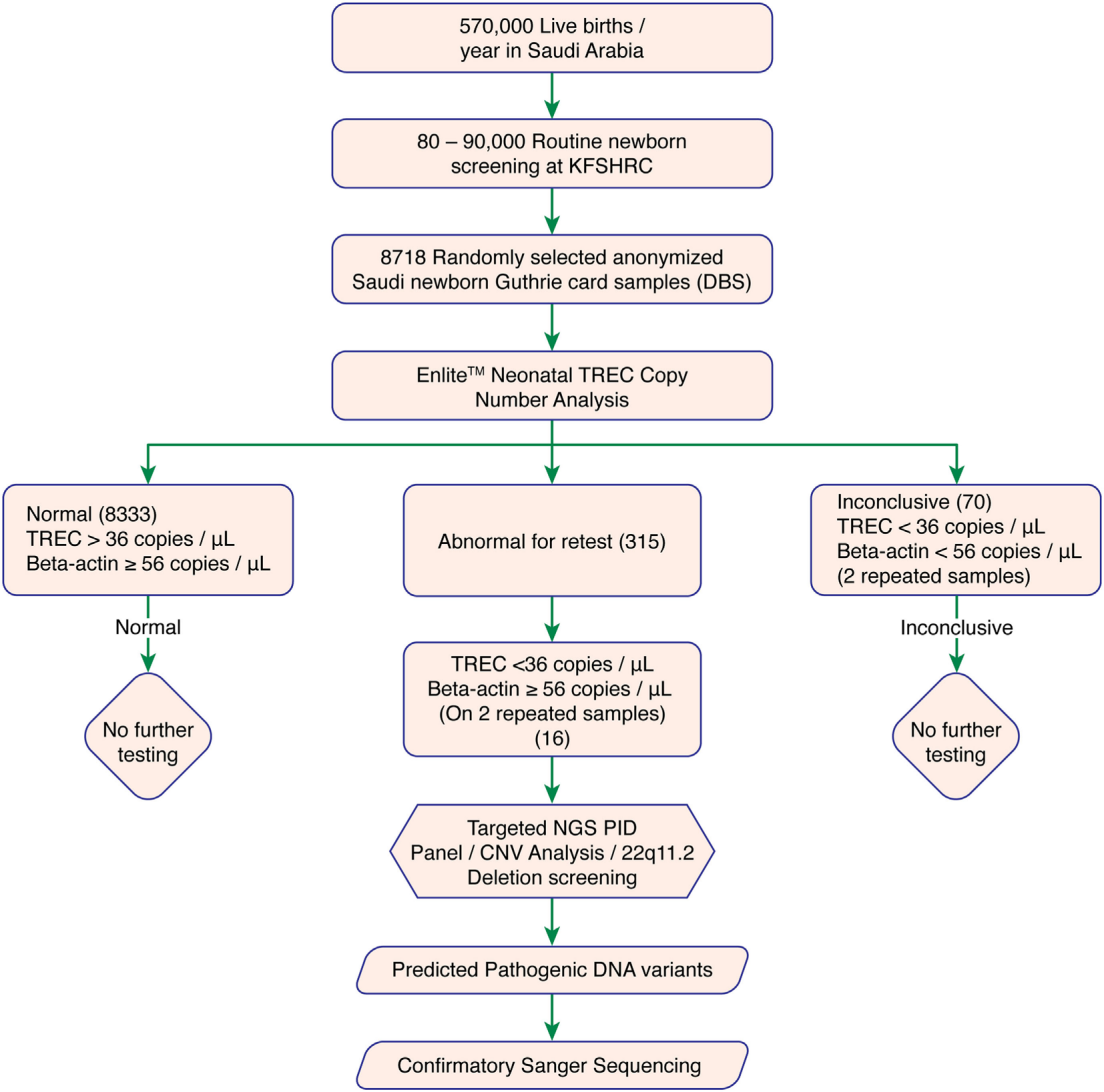
Experimental workflow. Abbreviations: KFSHRC, King Faisal Specialist Hospital & Research Center; DBS, dried blood spot; TREC, T cell receptor excision circle; NGS, Next generation sequencing; PID, primary immunodeficiency disease; qPCR, quantitative polymerase chain reaction.

We used DBS punches from 8,718 previously collected, anonymized Guthrie cards. A cutoff of ≥36 TRECs/μL and Beta-actin copy of ≥56 copies/μL was determined as normal. Failed DBS samples were repeated. Samples with persistently low TRECs and normal Beta-actin copy number were subjected to 2nd tier testing (qPCR-based copy number analysis for 22q11.2 deletion) to rule out 22q11.2 deletion syndrome, and Proton-Ion Torrent (NGS)-based targeted primary immunodeficiency genes analysis. Experimental work flow is shown in Figure 1.

### TREC Assay

8,333 of 8,718 tested samples had an initial TREC value above the cutoff. 315 samples had an initial TREC or Beta-actin copy value below the cutoff, which led us to perform a second (repeat) TREC analysis (retest-rate 3.6%). Most of these samples (299 of 315, 95%) passed the second tier with a TREC copy number above the cutoff. Therefore, only 16 of 8,718 (0.18% or 1/544) samples had persistently low TREC counts below the cutoff. These samples were suspected of having T-cell deficiency and were subjected to further investigations (recall rate 0.18%). A total of 70 samples with combined low TREC and beta-actin copy number failed both initial and repeat testing and were, therefore, excluded (failure rate 0.8%). Table S2 in Supplementary Material showed samples with duplicate low TREC copy number.

### Mutations Detected by T-NGS PID Panel

All 16 samples with low TREC assay underwent T-NGS PID panel. A homozygous mutation in *AK2*, a compound heterozygous mutation in *JAK3*, and a homozygous mutation in *MTHFD1* were identified in three samples, respectively, and confirmed by Sanger sequencing. Details of the mutations, gestational age, and TREC level are shown in Table 1. The homozygous *AK2* mutation is a known Saudi mutation that has been previously reported to cause classical reticular dysgenesis (34). It is interesting to note that the recessive *JAK3* mutation was a compound heterozygous for a novel truncating and a rare deleterious mutation suggesting parental non-consanguinity. The *JAK3* and *MTHFD1* detected variants in samples 2 and 3 are novel.

**Table 1:** Mutations detected by T-next generation sequencing (NGS) primary immunodeficiency disease panel.

Sample	T cell receptor excision circle copies/μL (initial/repeat)	Gene	Mutation	Protein effect	Zygosity	Mutation type	Detected by T-NGS	Confirmed by Sanger
*SGP2017-00440*	6/0	*AK2*	NM_001625:exon6:c.524G > C	p.R175P	Homozygous	SNV (29)	YES	YES

*SGP2017-00442*	3/3	*JAK3*	NM_000215:exon10:c.1275T > A	p.Y425X	Compound heterozygous	SNV^a^ (novel)	YES	YES
			NM_000215:exon3:c.308G > A	p.R103H		(rare, 8.129e-6; deleterious)	YES	YES

*SGP2017-00425*	34/36	*MTHFD1*	NM_005956:exon24:c.2404G > A	p.V802I	Homozygous	SNV (rare, 7.326e-5; deleterious)	YES	YES

*SNV, single nucleotide variant*.

*^a^Very rare deleterious SNVs (http://gnomad.broadinstitute.org/) which have never been reported as homozygous*.

### Copy Number Variants

Copy number variation analyses were carried out for each of the low TREC samples to rule out large insertions or deletions. No large homozygous deletions were detected.

### Chr.22q11.2 Copy Number/Deletion Analysis

Among the 16 samples with low TREC copy number, two samples with 22q11.2 deletion were suspected by qPCR. No further confirmatory testing was performed.

### Prematurity

692 out of the 8,718 samples were from preterm newborns (<37 weeks gestation) representing 8% of all samples recruited. Three out of the sixteen samples with low TREC assay belonged to preterm newborn (19%).

### SCID Incidence in Saudi Arabia

Three suspected SCID cases were identified after screening 8,718 Saudi newborn Guthrie card samples based on EnLite™ Neonatal TREC assay followed by T-NGS PID Panel and confirmatory Sanger sequencing. These results indicate a frequency of SCID of 1/2,906 live births in Saudi Arabia. Further confirmatory testing using flow cytometry and clinical correlation were not possible per protocol.

## Discussion

Severe combined immunodeficiency disease epidemiology from worldwide registries showed a geographical and racial variation. For multiple socio-cultural factors, consanguineous marriages are a common practice in the Middle East and Northern Africa (MENA) region with an overall incidence ranging between 20 and 50% (27, 35–40). This led to the abundance of autosomal recessive diseases among various MENA populations. Published data from highly consanguineous populations such as Kuwait showed an estimated incidence of 1/7,500 live birth for combined immunodeficiency diseases (41) and data from Saudi Arabia showed that SCID is the commonest PID (33%) (21). However, the true incidence of SCID among Saudi population was unknown. This study had provided the first population-based incidence of SCID at 1 in 2,906 Saudi live births, which is (20×) higher than the incidence reported from USA NBS programs (19). Although in this pilot study the sample size was relatively small to ascertain this high incidence, we believe that it is representative of the population of Saudi Arabia since our NBS services cover all regions of the country. A large scale NBS for SCID in Saudi Arabia will be needed to confirm this finding. This high incidence will increase the cost effectiveness ratio of implementing SCID NBS in such highly consanguineous population. This is possible provided that the health policy makers recognize the real incidence and the seriousness of such disease and provide all required resources to manage it.

Severe combined immunodeficiency disease is caused by mutations in one of several genes including *IL2RG, JAK3, DCLRE1C, RAG1, RAG2, IL7R, ADA, CD3D, CD3E, CD3Z, DOCK2, AK2*, and *TTC7A* (23, 42, 43). Additional genetic defects in *MTHFD1, RMRP, CORO1A, PNP, DOCK8, ATM*, and *BCL11B*, among others also cause combined immunodeficiencies, which can be detected by low TREC copy number analysis (23). TREC NBS has revealed a small number of infants with non-SCID T cell (idiopathic) lymphopenia for which no apparent cause was identified (44). Newly developed and commercially available SCID NGS gene panels are available for clinicians to order on newborns with low TREC assay. However, such panels will miss many of the possible causing syndromes in addition to non-SCID causing defects. Whole-exome sequencing (without concomitant copy number analysis) will miss diagnoses such as 22q11.2 deletion syndrome, trisomy 21, deletions, and other cytogenetic syndromes. Therefore, it is important to have a short list of all possible syndromes and genetic defects that will prompt further follow-up investigations.

Dried blood spots are potential resources for genetic and genomic analysis. Recent studies showed that sufficient DBS DNA can be extracted and used for NGS to perform whole exome sequencing (WES) and whole genome sequencing (WGS) without genome amplification (45). The use of next-generation sequencing has the potential to be integrated in SCID NBS programs to facilitate and accelerate genetic testing and final diagnosis of affected newborns. However, appropriate utilization of these technologies will require the capacity to manage and interpret large amounts of genetic data. Implementing such testing will also raise several questions about the ability of clinicians to interpret and effectively communicate the generated genetic data (46, 47). A more focused, clinically driven NGS gene panel-based analysis covering entire genes (to capture known and novel variants) will be more appropriate for initial screening for mutations that can explain the low TREC counts and will reduce many of the complexities associated with WES or WGS. Targeted NGS gene panel assay has a rapid turn-around time and the lists of potential candidate variants generated is much shorter compared to WES or WGS. This is expected to facilitate result interpretation and reduce the risk of incidental findings. WES or WGS can be applied to unsolved cases for new gene discovery. All novel variants, even in known PID genes will require functional and experimental validation to prove that they are actually deleterious and will remain the benchmark for causal association with a disease phenotype.

Most newborns found to have low TREC counts will undergo extensive follow-up genetic testing to identify the possible underlying immune defect. This approach has revealed a broad range of conditions with a wide spectrum of clinical severity in which immune deficiency may occur, some of which were not previously appreciated to cause a significant immunodeficiency phenotype. Currently, the post-NBS genetic testing for SCID related molecular defects is initiated after performing full clinical and immunological assessments. However, the development of high-throughput DNA sequencing technologies has revolutionized the genetic testing approach potentially allowing early initiation of genetic testing, even before starting immunological work up, which may lead to a more efficient and rapid identification of the genetic basis of inherited inborn errors of immunity. An ideal NBS NGS PID gene panel will include all PID genes known to be associated with low TREC counts. This list can be easily revised on a regular basis to include newly discovered genes. Therefore, we believe that this combined approach (TREC followed by NGS-based PID gene analysis) can be used efficiently in the screening and diagnosis of newborns with low TREC counts.

In this study, we elected to use a high TREC copies cutoffs (36 copies/μL) since we had no access to patients to perform further immunological testing. In the USA and Europe, lower cutoffs had been implemented in routine NBS (48). Table 2 demonstrates the performance of this assay in Saudi newborns based on various TREC copies cutoffs scenarios. At 36 copies/μL and based on a population of 570,000 newborns, an estimated 1,026 newborns will need referral for clinical and immunological evaluations and hence a high number of newborns will be called for retesting creating unnecessary anxiety among parents and increase in health-care expenditures. A lower cutoff, at 20 copies/μL, is expected to identify all classical SCID cases and result in a more reasonable number of clinical referrals (171). However, an atypical SCID and other T cell lymphopenia can be missed. Case in point, the sample with MTHFD1 deficiency would not have been identified at this low cutoff (Table 1). MTHFD1 deficiency is a recently discovered metabolic disorder characterized by severe combined immunodeficiency, megaloblastic anemia, and neurological deficits (49, 50). A larger, more comprehensive pilot study supported by clinical outcomes of various cutoff limits should determine the final TREC cutoff level to be used in routine NBS that will ensure the identification of all babies with typical SCID.

**Table 2:** Comparison of number of needed referrals for confirmatory studies for severe combined immunodeficiency disease based on T cell receptor excision circle (TREC) concentration cutoffs.

	35 copies/μL	30 copies/μL	25 copies/μL	20 copies/μL	15 copies/μL
Number and (%) positives after first TREC assay	315 (3.6%)	263 (3%)	199 (2.3)	161 (1.8)	124 (1.4)
Number and (%) presumptive positives (after repeated TREC assay)	16 (0.18)	12 (0.14)	8 (0.09)	3 (0.03)	3 (0.03)
Total number of referrals per annum (KSA)^a^	1,026	798	513	171	171
Total number of referrals per annum (UK) (14)^b^	1,680	554	420	210	NA
Total number of referrals per annum (Netherlands) (18)^c^	1,209	946	403	140	NA

*Numbers were calculated based on a total of 570,000^a^, 700,000^b^, and 178,000^c^ annual live births in KSA, UK, and Netherlands respectively*.

*NA, not available*.

The economical evaluation of the cost effectiveness of novel technologies for neonatal screening is a challenging task, as various factors determine their outcome. Extensive genetic testing is already part of the routine work up of symptomatic newborns. It is now clear that the targeted NGS approach is a cost-effective and more rapid alternative to Sanger sequencing especially for the evaluation of large genes and multi-genetic disorders (e.g., Omenn syndrome) (28). The consumables cost for our targeted NGS method is approximately $250 per sample based upon multiplexing of approximately 40 samples per run. A comprehensive cost effectiveness study of implementing additional NGS-based testing will be needed before making it part of the TREC-based NBS program.

In summary, this TREC-based SCID NBS pilot study provides the first evidence of high incidence of SCID among the Saudi population and demonstrates the feasibility of using T-NGS PID panel from Guthrie card DBSs as a new reliable, rapid, and cost-effective mutation screening method for newborns with low TREC counts.

## Ethics Statement

All research involving Guthrie card samples was approved by the institutional review board at King Faisal Specialist Hospital & Research Center (KFSHRC), RAC:2130-027and supported by the Kingdom of Saudi Arabia National Science, Technology and Innovation Plan’s Strategic Technologies Grant KACST: 13-BIO 755-20 under the King Abdulaziz City for Science and Technology (Riyadh, Kingdom of Saudi Arabia).

## Author Contributions

HA-M and MD designed the study, analyzed data, and prepared the manuscript. AH participated in the design of the study and data analysis. GA-D and AJ performed the screening for TREC and beta-actin and collected the results. FE and MB assisted in data analysis. DM and BM performed the NGS. MA assisted in bioinformatics analysis. All authors participated and approved the final version for submission.

## Conflict of Interest Statement

The authors declare that the research was conducted in the absence of any commercial or financial relationships that could be construed as a potential conflict of interest.

## Footnotes

^1^www.appliedbiosystems.com (Accessed: March 27, 2018).

^2^https://tools.thermofisher.com/content/sfs/manuals/cms_062368.pdf (Accessed: March 27, 2018).

## Abbreviations

DBS, dried blood spot; NBS, newborn screening; NGS, next generation sequencing; PID, primary immunodeficiency disease; qPCR, quantitative polymerase chain reaction; SCID, severe combined immunodeficiency disease; SNP, single nucleotide polymorphism; SNV, single nucleotide variant; TREC, T cell receptor excision circle; CNV, copy number variation; WES, whole exome sequencing; WGS, whole genome sequencing.

## References

[1] Notarangelo LD. Primary immunodeficiencies. J Allergy Clin Immunol (2010) 125(2 Suppl 2):S182–94. 10.1016/j.jaci.2009.07.053 20042228

[2] van der Burg M Gennery AR Educational paper. The expanding clinical and immunological spectrum of severe combined immunodeficiency. Eur J Pediatr (2011) 170(5):561–71. 10.1007/s00431-011-1452-3 21479529 PMC3078321

[3] Fischer A Notarangelo LD Neven B Cavazzana M Puck JM Severe combined immunodeficiencies and related disorders. Nat Rev Dis Primers (2015) 1:15061 10.1038/nrdp.2015.61 27189259

[4] Chinn IK Shearer WT. Severe combined immunodeficiency disorders. Immunol Allergy Clin North Am (2015) 35(4):671–94. 10.1016/j.iac.2015.07.002 26454313

[5] Myers LA Patel DD Puck JM Buckley RH. Hematopoietic stem cell transplantation for severe combined immunodeficiency in the neonatal period leads to superior thymic output and improved survival. Blood (2002) 99(3):872–8. 10.1182/blood.V99.3.872 11806989

[6] Brown L Xu-Bayford J Allwood Z Slatter M Cant A Davies EG Neonatal diagnosis of severe combined immunodeficiency leads to significantly improved survival outcome: the case for newborn screening. Blood (2011) 117(11):3243–6. 10.1182/blood-2010-08-300384 21273302

[7] Pai SY Logan BR Griffith LM Buckley RH Parrott RE Dvorak CC Transplantation outcomes for severe combined immunodeficiency, 2000-2009. N Engl J Med (2014) 371(5):434–46. 10.1056/NEJMoa1401177 25075835 PMC4183064

[8] Wilson JM Jungner YG [Principles and practice of mass screening for disease]. Bol Oficina Sanit Panam (1968) 65(4):281–393. 4234760

[9] Borte S von Dobeln U Hammarstrom L. Guidelines for newborn screening of primary immunodeficiency diseases. Curr Opin Hematol (2013) 20(1):48–54. 10.1097/MOH.0b013e32835a9130 23108220

[10] Douek DC McFarland RD Keiser PH Gage EA Massey JM Haynes BF Changes in thymic function with age and during the treatment of HIV infection. Nature (1998) 396(6712):690–5. 10.1038/25374 9872319

[11] Chan K Puck JM. Development of population-based newborn screening for severe combined immunodeficiency. J Allergy Clin Immunol (2005) 115(2):391–8. 10.1016/j.jaci.2004.10.012 15696101

[12] Morinishi Y Imai K Nakagawa N Sato H Horiuchi K Ohtsuka Y Identification of severe combined immunodeficiency by T-cell receptor excision circles quantification using neonatal Guthrie cards. J Pediatr (2009) 155(6):829–33. 10.1016/j.jpeds.2009.05.026 19628217

[13] Routes JM Grossman WJ Verbsky J Laessig RH Hoffman GL Brokopp CD Statewide newborn screening for severe T-cell lymphopenia. JAMA (2009) 302(22):2465–70. 10.1001/jama.2009.1806 19996402

[14] Adams SP Rashid S Premachandra T Harvey K Ifederu A Wilson MC Screening of neonatal UK dried blood spots using a duplex TREC screening assay. J Clin Immunol (2014) 34(3):323–30. 10.1007/s10875-014-0007-6 24668299

[15] Audrain M Thomas C Mirallie S Bourgeois N Sebille V Rabetrano H Evaluation of the T-cell receptor excision circle assay performances for severe combined immunodeficiency neonatal screening on Guthrie cards in a French single centre study. Clin Immunol (2014) 150(2):137–9. 10.1016/j.clim.2013.11.012 24412905

[16] Somech R Lev A Simon AJ Korn D Garty BZ Amariglio N Newborn screening for severe T and B cell immunodeficiency in Israel: a pilot study. Isr Med Assoc J (2013) 15(8):404–9. 24079059

[17] Chien YH Chiang SC Chang KL Yu HH Lee WI Tsai LP Incidence of severe combined immunodeficiency through newborn screening in a Chinese population. J Formos Med Assoc (2015) 114(1):12–6. 10.1016/j.jfma.2012.10.020 25618583

[18] Blom M Pico-Knijnenburg I Sijne-van Veen M Boelen A Bredius RGM van der Burg M An evaluation of the TREC assay with regard to the integration of SCID screening into the Dutch newborn screening program. Clin Immunol (2017) 180:106–10. 10.1016/j.clim.2017.05.007 28487086

[19] Kwan A Abraham RS Currier R Brower A Andruszewski K Abbott JK Newborn screening for severe combined immunodeficiency in 11 screening programs in the United States. JAMA (2014) 312(7):729–38. 10.1001/jama.2014.9132 25138334 PMC4492158

[20] Al-Herz W Al-Mousa H. Combined immunodeficiency: the middle east experience. J Allergy Clin Immunol (2013) 131(3):658–60. 10.1016/j.jaci.2012.11.033 23321211

[21] Al-Saud B Al-Mousa H Al Gazlan S Al-Ghonaium A Arnaout R Al-Seraihy A Primary immunodeficiency diseases in Saudi Arabia: a tertiary care hospital experience over a period of three years (2010-2013). J Clin Immunol (2015) 35(7):651–60. 10.1007/s10875-015-0197-6 26395454

[22] Al-Herz W Aldhekri H Barbouche MR Rezaei N. Consanguinity and primary immunodeficiencies. Hum Hered (2014) 77(1–4):138–43. 10.1159/000357710 25060276

[23] Jyonouchi S Jongco AM Puck J Sullivan KE. Immunodeficiencies associated with abnormal newborn screening for T Cell and B cell lymphopenia. J Clin Immunol (2017) 37(4):363–74. 10.1007/s10875-017-0388-4 28353166

[24] Dasouki M Okonkwo KC Ray A Folmsbeel CK Gozales D Keles S Deficient T cell receptor excision circles (TRECs) in autosomal recessive hyper IgE syndrome caused by DOCK8 mutation: implications for pathogenesis and potential detection by newborn screening. Clin Immunol (2011) 141(2):128–32. 10.1016/j.clim.2011.06.003 21763205 PMC4210456

[25] Mallott J Kwan A Church J Gonzalez-Espinosa D Lorey F Tang LF Newborn screening for SCID identifies patients with ataxia telangiectasia. J Clin Immunol (2013) 33(3):540–9. 10.1007/s10875-012-9846-1 23264026 PMC3591536

[26] King JR Hammarstrom L Newborn screening for primary immunodeficiency diseases: history, current and future practice. J Clin Immunol (2018) 38(1):56–66. 10.1007/s10875-017-0455-x 29116556 PMC5742602

[27] Al-Mousa H Al-Saud B. Primary immunodeficiency diseases in highly consanguineous populations from middle east and north Africa: epidemiology, diagnosis, and care. Front Immunol (2017) 8:678. 10.3389/fimmu.2017.00678 28694805 PMC5483440

[28] Al-Mousa H Abouelhoda M Monies DM Al-Tassan N Al-Ghonaium A Al-Saud B Unbiased targeted next-generation sequencing molecular approach for primary immunodeficiency diseases. J Allergy Clin Immunol (2016) 137(6):1780–7. 10.1016/j.jaci.2015.12.1310 26915675

[29] Al-Odaib AN Abu-Amero KK Ozand PT Al-Hellani AM. A new era for preventive genetic programs in the Arabian peninsula. Saudi Med J (2003) 24(11):1168–75. 14647548

[30] Alfadhel M Al Othaim A Al Saif S Al Mutairi F Alsayed M Rahbeeni Z Expanded newborn screening program in Saudi Arabia: incidence of screened disorders. J Paediatr Child Health (2017) 53(6):585–91. 10.1111/jpc.13469 28337809

[31] Food, Drug Administration HHS. Medical devices; immunology and microbiology devices; classification of the newborn screening test for severe combined immunodeficiency disorder. Final order. Fed Regist (2017) 82(208):50077–80. 29091371

[32] Routier FH Hounsell EF Rudd PM Takahashi N Bond A Hay FC Quantitation of the oligosaccharides of human serum IgG from patients with rheumatoid arthritis: a critical evaluation of different methods. J Immunol Methods (1998) 213(2):113–30. 10.1016/S0022-1759(98)00032-5 9692845

[33] Alsum Z Hawwari A Alsmadi O Al-Hissi S Borrero E Abu-Staiteh A Clinical, immunological and molecular characterization of DOCK8 and DOCK8-like deficient patients: single center experience of twenty-five patients. J Clin Immunol (2013) 33(1):55–67. 10.1007/s10875-012-9769-x 22968740

[34] Al-Zahrani D Al-Ghonaium A Al-Mousa H Al-Kassar A Roifman CM Skeletal abnormalities and successful hematopoietic stem cell transplantation in patients with reticular dysgenesis. J Allergy Clin Immunol (2013) 132(4):993–6. 10.1016/j.jaci.2013.04.055 23763981

[35] El-Mouzan MI Al-Salloum AA Al-Herbish AS Qurachi MM Al-Omar AA. Regional variations in the prevalence of consanguinity in Saudi Arabia. Saudi Med J (2007) 28(12):1881–4. 18060221

[36] Al-Awadi SA Moussa MA Naguib KK Farag TI Teebi AS el-Khalifa M Consanguinity among the Kuwaiti population. Clin Genet (1985) 27(5):483–6. 10.1111/j.1399-0004.1985.tb00236.x 4006273

[37] Hafez M El-Tahan H Awadalla M El-Khayat H Abdel-Gafar A Ghoneim M. Consanguineous matings in the Egyptian population. J Med Genet (1983) 20(1):58–60. 10.1136/jmg.20.1.58 6842535 PMC1048987

[38] Ben Halim N Ben Alaya Bouafif N Romdhane L Kefi Ben Atig R Chouchane I Bouyacoub Y Consanguinity, endogamy, and genetic disorders in Tunisia. J Community Genet (2013) 4(2):273–84. 10.1007/s12687-012-0128-7 23208456 PMC3666836

[39] Saadat M Ansari-Lari M Farhud DD. Consanguineous marriage in Iran. Ann Hum Biol (2004) 31(2):263–9. 10.1080/03014460310001652211 15204368

[40] Akbayram S Sari N Akgun C Dogan M Tuncer O Caksen H The frequency of consanguineous marriage in eastern Turkey. Genet Couns (2009) 20(3):207–14. 19852426

[41] Al-Herz W Notarangelo LD Sadek A Buckley R Consortium U. Combined immunodeficiency in the United States and Kuwait: comparison of patients’ characteristics and molecular diagnosis. Clin Immunol (2015) 161(2):170–3. 10.1016/j.clim.2015.07.013 26248333 PMC4869855

[42] Bousfiha A Jeddane L Al-Herz W Ailal F Casanova JL Chatila T The 2015 IUIS phenotypic classification for primary immunodeficiencies. J Clin Immunol (2015) 35(8):727–38. 10.1007/s10875-015-0198-5 26445875 PMC4659854

[43] Picard C Al-Herz W Bousfiha A Casanova JL Chatila T Conley ME Primary immunodeficiency diseases: an update on the classification from the international union of immunological societies expert committee for primary immunodeficiency 2015. J Clin Immunol (2015) 35(8):696–726. 10.1007/s10875-015-0201-1 26482257 PMC4659841

[44] Kwan A Puck JM. History and current status of newborn screening for severe combined immunodeficiency. Semin Perinatol (2015) 39(3):194–205. 10.1053/j.semperi.2015.03.004 25937517 PMC4433840

[45] Bassaganyas L Freedman G Vaka D Wan E Lao R Chen F Whole exome and whole genome sequencing with dried blood spot DNA without whole genome amplification. Hum Mutat (2018) 39(1):167–71. 10.1002/humu.23356 29067733 PMC5738671

[46] Tarini BA Goldenberg AJ. Ethical issues with newborn screening in the genomics era. Annu Rev Genomics Hum Genet (2012) 13:381–93. 10.1146/annurev-genom-090711-163741 22559326 PMC3625041

[47] Goldenberg AJ Sharp RR The ethical hazards and programmatic challenges of genomic newborn screening. JAMA (2012) 307(5):461–2. 10.1001/jama.2012.3643 22298675 PMC3868436

[48] van der Spek J Groenwold RH van der Burg M van Montfrans JM. TREC based newborn screening for severe combined immunodeficiency disease: a systematic review. J Clin Immunol (2015) 35(4):416–30. 10.1007/s10875-015-0152-6 25893636 PMC4438204

[49] Keller MD Ganesh J Heltzer M Paessler M Bergqvist AG Baluarte HJ Severe combined immunodeficiency resulting from mutations in MTHFD1. Pediatrics (2013) 131(2):e629–34. 10.1542/peds.2012-0899 23296427

[50] Field MS Kamynina E Watkins D Rosenblatt DS Stover PJ. Human mutations in methylenetetrahydrofolate dehydrogenase 1 impair nuclear de novo thymidylate biosynthesis. Proc Natl Acad Sci U S A (2015) 112(2):400–5. 10.1073/pnas.1414555112 25548164 PMC4299200

