# Supplementary material for: High Incidence of Severe Combined Immunodeficiency Disease in Saudi Arabia Detected Through Combined T Cell Receptor Excision Circle and Next Generation Sequencing of Newborn Dried Blood Spots
Source: Front Immunol. 2018 Apr 16;9:782. doi: 10.3389/fimmu.2018.00782 (PMC5911483; doi:10.3389/fimmu.2018.00782)
Supplement: Supplementary file 1 [file table_1.PDF]

# Supplementary table 1: The list of PID genes (265) included in PID T-NGS Panel

| | | | | | | | | | | | | | | |
|---|---|---|---|---|---|---|---|---|---|---|---|---|---|---|
| ACP5 | C1QA | CASP8 | CFB | CYBB | FPR1 | IL10RA | IRF8 | MBL2 | NHEJ1 | PRKCD | RNASEH2B | STAT3 | TINF2 | UNC13D |
| ACTB | C1QB | CCBE1 | CFD | DCLRE1C | G6PC3 | IL10RB | ISG15 | MCM4 | NHP2 | PRKDC | RNASEH2C | STAT4 | TLR3 | UNC93B1 |
| ADA | C1QC | CD19 | CFH | DKC1 | GATA2 | IL12B | ITCH | MEFV | NLRC4 | PSMB8 | RNF168 | STAT5B | TMC6 | UNG |
| ADAM17 | C1R | CD247 | CFHR1 | DNMT3B | GFI1 | IL12RB1 | ITGB2 | MKL1 | NLRP12 | PSTPIP1 | RNF31 | STIM1 | TMC8 | USB1 |
| ADAR | C1S | CD27 | CFHR3 | DOCK2 | GPI | IL12RB2 | ITK | MLPH | NLRP3 | PTPN6 | RPSA | STK4 | TNFRSF13B | VPS13B |
| AICDA | C2 | CD3D | CFI | DOCK8 | HAX1 | IL17F | JAGN1 | MRE11A | NOD2 | PTPRC | RTEL1 | STX11 | TNFRSF13C | VPS45 |
| AIRE | C3 | CD3E | CFP | ELANE | ICOS | IL17RA | JAK3 | MS4A1 | NOP10 | RAB27A | SAMHD1 | STXBP2 | TNFRSF1A | WAS |
| AK2 | C4A | CD3G | CIB1 | EPCAM | IFIH1 | IL17RC | LAMTOR2 | MSH6 | NRAS | RAC2 | SBDS | TAP1 | TNFRSF4 | WIPF1 |
| AP3B1 | C4B | CD40 | CIITA | EPG5 | IFNG | IL1RN | LCK | MTHFD1 | ORAI1 | RAG1 | SERPING1 | TAP2 | TNFSF12 | WRAP53 |
| AP3D1 | C5 | CD40LG | CLEC7A | F12 | IFNGR1 | IL21 | LIG1 | MVK | PGM3 | RAG2 | SH2D1A | TAPBP | TPP2 | XIAP |
| APOL1 | C6 | CD46 | CORO1A | FADD | IFNGR2 | IL21R | LIG4 | MYD88 | PIGA | RBCK1 | SKIV2L | TAZ | TRAC | XRCC4 |
| ARTN | C7 | CD59 | CR2 | FAS | IGHG2 | IL2RA | LPIN2 | MYO5A | PIK3CD | RFX5 | SLC11A1 | TBK1 | TRAF3IP2 | ZAP70 |
| ATM | C8A | CD79A | CSF2RA | FASLG | IGHM | IL2RG | LRBA | NBN | PIK3R1 | RFXANK | SLC37A4 | TBX1 | TREX1 | ZBTB24 |
| BCL10 | C8B | CD79B | CTLA4 | FCGR3B | IGKC | IL6 | LRRC8A | NCF1 | PLCG2 | RFXAP | SMARCAL1 | TCF3 | TRNT1 | |
| BLM | C9 | CD81 | CTPS1 | FCN3 | IGLL1 | IL7R | LYST | NCF2 | PMS2 | RHOH | SP110 | TERC | TTC37 | |
| BLNK | CARD11 | CD8A | CTSC | FERMT3 | IKBKG | INO80 | MAGT1 | NCF4 | PNP | RLTPR | SPPL2A | TERT | TTC7A | |
| BLOC1S6 | CARD9 | CEBPE | CXCR4 | FOXN1 | IKZF1 | IRAK4 | MALT1 | NFKB2 | POLE | RMRP | STAT1 | TGFBR2 | TYK2 | |
| BTK | CASP10 | CECR1 | CYBA | FOXP3 | IL10 | IRF3 | MASP2 | NFKBIA | PRF1 | RNASEH2A | STAT2 | TICAM1 | UNC119 | |
