# Supplementary material for: High Incidence of Severe Combined Immunodeficiency Disease in Saudi Arabia Detected Through Combined T Cell Receptor Excision Circle and Next Generation Sequencing of Newborn Dried Blood Spots
Source: Front Immunol. 2018 Apr 16;9:782. doi: 10.3389/fimmu.2018.00782 (PMC5911483; doi:10.3389/fimmu.2018.00782)
Supplement: Supplementary file 2 [file table_2.PDF]

Supplementary table 2: all 16 samples with duplicate low TREC copy number

Sample Number	Gestational age (wks)	TREC Count (initial/repeat)
SGP2017-00414	38	35 / 28
SGP2017-00416	39	23 / 29
SGP2017-00418	38	26 /2 4
SGP2017-00425	37	34 / 36
SGP2017-00426	39	30 / 28
SGP2017-00427	40	23 / 25
SGP2017-00428	39	32 / 29
SGP2017-00430	35	29 / 28
SGP2017-00431	40	34 / 32
SGP2017-00432	27	19 / 34
SGP2017-00433	37	0 / 2
SGP2017-00434	40	10 / 9
SGP2017-00438	36	9 / 32
SGP2017-00440	38	6 / 0
SGP2017-00441	41	35 / 22
SGP2017-00442	38	3 / 3
